# Complete Genome Sequence of Klebsiella pneumoniae Myophage Menlow

**DOI:** 10.1128/MRA.00192-19

**Published:** 2019-04-25

**Authors:** Heather N. Newkirk, Lauren Lessor, Jason J. Gill, Mei Liu

**Affiliations:** a Center for Phage Technology, Texas A&M University, College Station, Texas, USA; Queens College

## Abstract

Klebsiella pneumoniae is an opportunistic pathogen that has become an increasing problem in nosocomial infections. Studying phages that infect K. pneumoniae may lead to improvements in phage therapeutics for treating these infections. Here, the full genome sequence of Menlow, a Vi01-like phage, is introduced and described.

## Abstract (precis)

Klebsiella pneumoniae is an opportunistic pathogen that has become an increasing problem in nosocomial infections. Studying phages that infect K. pneumoniae may lead to improvements in phage therapeutics for treating these infections.

## ANNOUNCEMENT

Klebsiella pneumoniae is a Gram-negative rod found in the normal flora of the mouth, skin, and intestines (1). Strains of K. pneumoniae producing the *bla*_KPC_ carbapenemase are resistant to a broad range of antibiotics and are of serious concern in hospital settings (2, 3). By identifying and characterizing phages that infect K. pneumoniae, new therapies and treatments may be discovered.

Phage Menlow was isolated from influent water at the Carter Creek Wastewater treatment plant in College Station, TX, against a carbapenemase-producing K. pneumoniae isolate of the sequence type 258 (ST258) lineage. Host bacteria were cultured on tryptic soy broth or agar (Difco) at 37°C with aeration. Phages were isolated and propagated by the soft agar overlay method (4). Phage genomic DNA was prepared using a modified Promega Wizard DNA cleanup kit protocol as described previously (5). Pooled indexed DNA libraries were prepared using the Illumina TruSeq Nano LT kit, and a sequence was obtained from the Illumina MiSeq platform using the MiSeq v2 500-cycle reagent kit following the manufacturer’s instructions, producing 447,621 paired-end reads for the index containing the phage genome. Reads were quality controlled in FastQC v0.11.5 (https://www.bioinformatics.babraham.ac.uk/projects/fastqc), edited with FastX Toolkit v0.0.14 (http://hannonlab.cshl.edu/fastx_toolkit/index.html), and assembled in SPAdes v3.5.0 (6). The genome was closed by PCR using primers (5′-CGATGAATCCCTCTCGTTCTT-3′ and 5′-CATGGGAGAGCTTCTTGTACTT-3′) facing off the ends of the assembled contig and Sanger sequencing of the resulting product, with the contig sequence manually corrected to match the resulting Sanger sequencing read. Protein-coding genes were predicted using GLIMMER v3.0 (7) and MetaGeneAnnotator v1.0 (8) with manual editing; tRNA genes were predicted with ARAGORN v2.36 (9). Protein functions were determined based on sequence homology detected by BLASTp v2.2.28 (10), and conserved domain searches were detected using InterProScan v5.15-5.40 (11). Nucleotide percent identity was determined by the progressiveMauve algorithm v2.4.0 (12). All analyses were conducted using default settings via Center for Phage Technology (CPT) Galaxy (13) and WebApollo (14) interfaces (cpt.tamu.edu).

The Menlow genome was assembled to 157,281 bp at 244.5-fold coverage and was predicted to encode 212 proteins and 5 tRNAs. Menlow has a coding density of 93% and a GC content of 46.4%, which is significantly lower than the GC content of K. pneumoniae (57%) (15). According to a BLASTp search, Menlow is largely syntenic with Vi01 (16), with 182 similar proteins at an E value of <10^−5^. Menlow shows 87.3% nucleotide similarity with another K. pneumoniae phage, 0507-KN2-1 (GenBank accession no. AB797215) (17). It was suggested that Menlow and other similar phages belong to a new genus called “Viunalikevirus” (18). Many genes involved in DNA replication and phage morphology were identified. A superinfection exclusion protein similar to that of phage P22 gp17 was identified (19). Many of the hypothetical novel genes identified are clustered around the genome’s 5 tRNAs. A putative tape measure protein was identified based on the length of the protein and the presence of the alphahelical character predicted by Jpred3 (20). The endolysin was identified as an *N*-acetylmuramidase-like lysozyme (InterPro accession no. IPR024408); however, there were no identifiable holins. A spanin complex could not be reliably identified. The TerL gene shows homology to those of phages that use headful packaging (21).

### Data availability.

The genome sequence of phage Menlow was deposited under GenBank accession no. MG428990. The associated BioProject, SRA, and BioSample accession numbers are PRJNA222858, SRR8788475, and SAMN11259833, respectively.
